# Synergy between parthenolide and arsenic trioxide in adult T-cell leukemia/lymphoma cells *in vitro*

**DOI:** 10.22038/ijbms.2020.40650.9610

**Published:** 2020-05

**Authors:** Hamideh Kouhpaikar, Mohammad Hadi Sadeghian, Houshang Rafatpanah, Mohaddeseh Kazemi, Mehrdad Iranshahi, Zahra Delbari, Faezeh Khodadadi, Hossein Ayatollahi, Fatemeh B. Rassouli

**Affiliations:** 1 Cancer Molecular Pathology Research Center, Department of Hematology and Blood Bank, Faculty of Medicine, Mashhad University of Medical Sciences, Mashhad, Iran; 2 Inflammation and Inflammatory Diseases Research Center, Faculty of Medicine, Mashhad University of Medical Sciences, Mashhad, Iran; 3 Department of Pharmacognosy and Biotechnology, Biotechnology Research Center, Faculty of Pharmacy, Mashhad University of Medical Sciences, Mashhad, Iran; 4 Novel Diagnostics and Therapeutics Research Group, Institute of Biotechnology, Ferdowsi University of Mashhad, Mashhad, Iran

**Keywords:** Adult T-cell, Leukemia/Lymphoma, Arsenic trioxide, In vitro, Parthenolide, PCR

## Abstract

**Objective(s):**

Adult T-cell leukemia/lymphoma (ATLL) is an aggressive lymphoid malignancy with low survival rate and distinct geographical distribution. In search for novel chemotherapeutics against ATLL, we investigated the combinatorial effects of parthenolide, a sesquiterpene lactone with valuable pharmaceutical activities, and arsenic trioxide (ATO) *in vitro*.

**Materials and Methods:**

MT2 cells, an ATLL cell line, were treated with increasing concentrations of parthenolide (1.25, 2.5, and 5 μg/ml) and ATO (2, 4, 8, and 16 µM) to determine their IC_50_. Then, cells were treated with a combination of sub-IC_50_ concentrations of parthenolide (1 μg/ml) and ATO (2 µM) for 72 hr. Cell viability and cell cycle changes were assessed by Alamar blue and PI staining, respectively. To understand the mechanisms responsible for observed effects, expression of *CD44, NF-κB (REL-A), BMI-1*, and *C-MYC* were investigated by real-time PCR.

**Results:**

Assessment of cell viability indicated that parthenolide significantly increased the toxicity of ATO, as confirmed by accumulation of MT2 cells in the sub G1 phase of the cell cycle. Moreover, molecular analysis revealed significant down-regulation of *CD44, NF-κB (REL-A), BMI-1*, and *C-MYC* upon combinatorial administration of parthenolide and ATO in comparison with relevant controls.

**Conclusion:**

Taken together, present results showed that parthenolide significantly enhanced the toxicity of ATO in MT2 cells. Therefore, the future possible clinical impact of our study could be combinatorial use of parthenolide and ATO as a novel and more effective approach for ATLL.

## Introduction

Adult T-cell leukemia/lymphoma (ATLL) is an uncommon and aggressive lymphoid malignancy that is associated with human T-cell leukemia virus type1 (HTLV-1). It has been estimated that 10–20 million people are infected with HTLV-1, and endemic regions include Central Africa, South America, the Caribbean basin, Iran, South-Western Japan, and Melanesia (1, 2). Among HTLV-1 infected patients, approximately 3–5% develop ATLL in four categories: acute (60%), lymphomatous (20%), chronic (15%), and smoldering (5%) types (3, 4).

In recent years, survival of ATLL patients has been improved by introduction of multiagent chemotherapy, antiviral therapies, and allogeneic hematopoietic stem cell transplantation, as well as advances in supportive care (5). Among all chemotherapeutic regimes, high response rates were observed after coadministration of interferon (IFN-α) and antiviral agent zidovudine, and also interferon-alfa (IFN-α) and arsenic trioxide (ATO), especially in the acute ATLL patients (6). Nevertheless, ATLL relapses in most cases partially due to intrinsic drug resistance mediated by p-glycoprotein (p-gp) and anti-apoptotic proteins (7). 

Parthenolide, first isolated from medicinal plant *Tanacetum parthenium,* is a sesquiterpene lactone of the germacranolide class with a wide range of pharmaceutical activities such as anti-inflammatory and anti-cancer effects (8, 9). Parthenolide contains an alpha-methylene-gamma-lactone ring through which it interacts with nucleophilic sites of multiple biological targets and induces its various effects. Low solubility of parthenolide in water is a physico-chemical property that limits biological applications of this compound. However, a new approach has been proposed for synthesis of parthenolide derivatives with better solubility and higher potency (10). *In vitro* studies demonstrated anti-proliferative activity of parthenolide in the prostate, pancreatic and colorectal cancer cells (11-13), and also multiple myeloma and acute myelogenous leukemia cells (14). In addition, synergistic effects of parthenolide and chemotherapy drugs have been reported in breast carcinoma and colorectal adenocarcinoma cells (15, 16). Suppression of Nuclear factor kappa beta (NFκB) and Signal transducer and activator of transcription 3 (STAT3) signaling pathways, inhibition of A mitogen-activated protein kinase (MAPK) activity and induction of mitochondrial dysfunction are a number of mechanisms introduced for parthenolide anticancer effects (9). 

Poor outcome of current chemotherapy modalities in ATLL has made it crucial to investigate novel approaches with higher efficacy. Although anticancer and synergic effects of parthenolide have been demonstrated in various cell types, pharmaceutical effects of parthenolide, alone or in combination with other chemicals, have not as yet been reported in ATLL. Accordingly, we investigated whether combination of parthenolide with ATO could improve cytotoxicity in HTLV-1-infected cells. In this regard, cell viability was assessed using Alamar blue, cell cycle was analyzed by PI staining and flowcytometry, and expression patterns of CD44*, NF-κB* (*REL-A*), BMI-1*, *and* C-MYC *were investigated by real-time polymerase chain reaction (PCR).

## Materials and Methods

***Chemicals and reagents***

Parthenolide was obtained from Euroasia (China). ATO, Alamar blue, and propidium iodide (PI) were purchased from Sigma Aldrich (Germany). Dimethylsulfoxide (DMSO) and Triton X-100 were from Merck (Germany). RPMI 1640 was obtained from Biosera (France) and Fetal Bovine Serum (FBS), penicillin/streptomycin, and L-glutamine were supplied by Gibco (Scotland). Tripure was from Roche (Germany) and M-MuLV reverse transcriptase was from Thermo Scientific (USA), while the SYBR green mix was purchased from Takara (Japan).

***Cell treatment and viability assay***

The MT-2 cell line (lymphoma cells infected with HTLV-I derived by co-cultivating normal human cord leukocytes and human leukemic T-cells) was donated by Inflammation and Inflammatory Diseases Research Center, Faculty of Medicine, Mashhad University of Medical Sciences (generous gift from Prof. Houshang Rafatpanah). MT2 cells were maintained in RPMI 1640 supplemented with 10% FBS, 1% (W/V) penicillin/streptomycin, and 1% L-glutamine, and incubated at 37 ^°^C in 5% CO_2_. To prepare different concentrations of parthenolide, 2 mg of the crystal was dissolved in100 µl DMSO and diluted with complete culture medium, while equal amount of DMSO in all parthenolide concentrations (0.2% v/v) was considered as the control treatment. Half maximal inhibitory concentration (IC_50_) of parthenolide and ATO were determined upon treatment of MT2 cells (5 × 10^4^ cells/well) with increasing concentrations of parthenolide (1, 2.5, and 5µg/ml) and ATO (2, 4, 8, and 16 µM) for 24, 48, and 72 hr. Then, cells were treated with a combination of sub-IC_50_ concentrations of parthenolide (0.5, 0.75, and 1 µg/ml) and ATO (0.5, 1, and 2 µM) for 72 hr.

To assess the viability of cells, Alamar blue (0.1 mg/ml) was added (20 µl/well) by the end of each time point and cells were incubated at 37 °C for 2 hr. Then, absorbance was measured at 600 nm using a microplate reader (Epoch, USA) and cell viability (%) was calculated using the following equation: 100-((AT-AU)/(AB-AU)×100), in which AT and AU were absorbance of treated and untreated cells, respectively, and AB was absorbance of blank control.

To determine drug interactions in our combinatorial treatment, the Combination Index (CI) was calculated, which evaluates synergism or antagonism between drugs; A value of CI > 1 means antagonism, CI = 1 shows additive, and CI < 1 indicates synergism (17).

***Cell cycle analysis***

Changes induced in the cell cycle after combinatorial use of parthenolide and ATO were studied after PI staining. Briefly, MT2 cells in each treatment were collected and washed with cold PBS containing 5% FBS. Then, cell pellets were resuspended in a hypotonic buffer containing 100 μg/ml PI, 0.1% sodium citrate, and 0.1% Triton X-100, incubated for 30 min at 37 ^°^C in the dark, and analyzed by flowcytometry (BD FACSCalibur) using FL2 filter. 

***Gene expression studies***

Real-time PCR was performed to study the expression pattern of four candidate genes upon coadministration of parthenolide and ATO. To do so, at first the total cellular RNA was extracted from treated cells and their relevant controls using Tripure (Roche, Germany) followed by synthesis of cDNAs by random hexamer, dNTPs, and M-MuLV reverse transcriptase according to the manufacturer’s protocol. Real-time PCR was conducted in Rotor-Gene 6000 detection system (Qiagen, Germany) using the SYBR green mix and primers listed in Table 1 for CD44, BMI-1, and *C-MYC* genes, while a TaqMan probe and specific primers were used for *NF-κB* (*REL-A*). In all analyses, beta-2 microglobulin transcripts were considered as the internal control. PCR cycling conditions were 94 ^°^C for 2 min, [94 ^°^C for 15 sec, 60 ^°^C for 30 sec, 72 ^°^C for 30 sec] (40 cycles) for CD44, BMI-1, and *C-MYC*, and 95 ^°^C for 2 min, [95 ^°^C for 15 sec, 60 ^°^C for 30 sec, 72 ^°^C for 30 sec] (45 cycles) for *NF-κB* (*REL-A*).

***Statistical analysis***

The statistical signiﬁcance was assessed by one way ANOVA and Tukey multiple comparisons test using Graph pad prism version 6.07 and SPSS version 16. In addition, CI was calculated using the Compusyn software package and results of flowcytometry were analyzed by Win-MDI version 2.8. All data were presented as mean ± SD and *P*-values less than 0.05, 0.001, and 0.0001 were considered signiﬁcant for all comparisons.

## Results

***Inhibitory effects of parthenolide and ATO on the viability of MT-2 cells***

**Figure 1:**
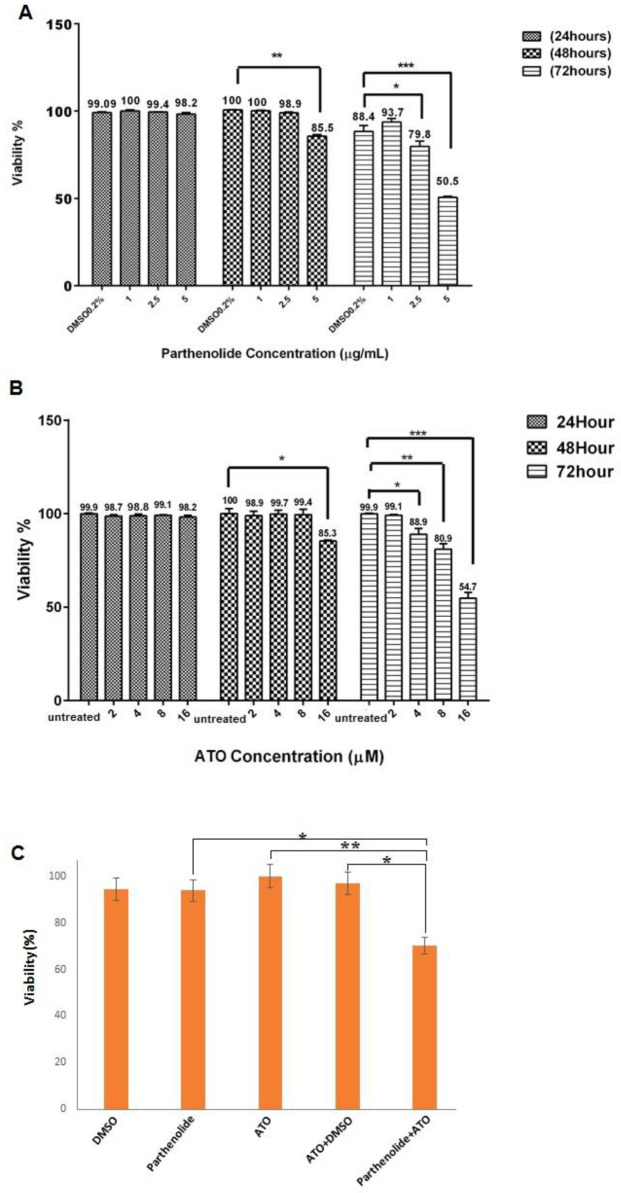
Cytotoxicity of parthenolide and ATO, alone or in combination, in MT2 cells. Cells were treated with various concentrations of parthenolide for 24, 48, and 72 hr, and IC50 of parthenolide was determined as 5 µg/ml after 72 hr (A). After treatment of MT-2 cells with various concentrations of ATO for 24, 48, and 72 hr, its IC50 was determined as >16 µM (B). Combinatorial use of parthenolide and ATO significantly reduced cell viability in comparison with other treatments (C)

As indicated in Figures 1 A and B, parthenolide and ATO did not induce significant toxic effects on MT2 cells after 24 hr. Nevertheless, 48 hr after treatment of cells with the highest concentrations of each agent, a significant decrease in cell viability was detected. However, IC_50_ of parthenolide and ATO was only determined after 72 hr, which was 5 µg/ml for parthenolide and >16 μM for ATO. To note, cells treated with 0.2% DMSO were considered as relevant control for all treatments including parthenolide.

***Combination of parthenolide and ATO induced more toxic effects on MT2 cells***

After determination of IC_50_ values, we investigated the combinatorial effects of parthenolide and ATO. In this regard, cells were treated for 72 hr with parthenolide and ATO alone and combination in concentrations lower than their IC_50_. Reduction in the cell viability after combination of parthenolide and ATO was significantly greater than that for parthenolide and ATO alone (0.001). As shown in Figure 1C, cytotoxicity of 2 µM ATO was increased by 1 µg/ml parthenolide up to 26.5%. CI values (Table 2) also indicated synergistic effects in different combinations of parthenolide and ATO.

***Combination of parthenolide and ATO induced cell cycle changes***

**Figure 2:**
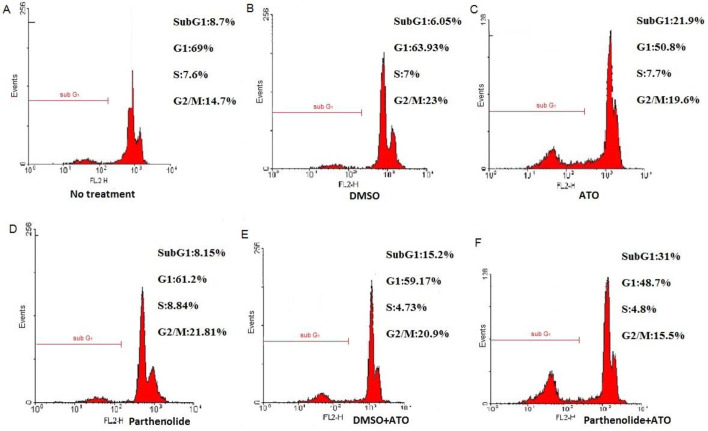
MT2 cell cycle analysis by PI staining. Untreated cells (A), cells treated with 0.2% DMSO (B), 2 µM ATO (C), 1 µg/ml parthenolide (D), 0.1% DMSO + 2 µM ATO (E), and 1 µg/ml parthenolide + 2 µM ATO (F) for 72 hr. Sub-G1 peak, as an indicative of apoptotic cells, was specifically induced after parthenolide + ATO combination

To determine whether combinatorial treatment of cells with parthenolide and ATO was associated with cell cycle changes, DNA content of MT2 cells was analyzed by flowcytometry. As presented in Figure 2, in untreated MT2 cells and cells treated with parthenolide and its relevant DMSO control 9%, 6.3%, and 8.1% of cells were detected in the sub G_1_ phase of the cell cycle, respectively. However, this amount increased up to 21.5% after ATO administration. Moreover, upon combinatorial treatment with parthenolide and ATO, 31.6% of cells were presented in the sub G_1_ phase, which was higher than that in DMSO and ATO treatment (15.1%). 

***Parthenolide and ATO decreased CD44, NF-*** ***κB*** ***(REL-A), and BMI-1 expression***

**Figure 3:**
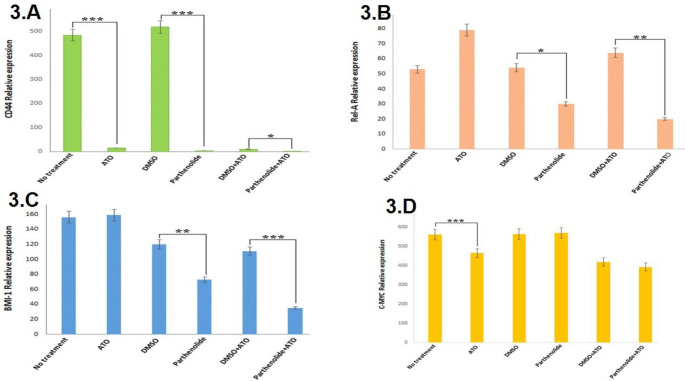
Real-time PCR analysis of CD44 (A), NF-κB (REL-A) (B), BMI-1 (C), and C-MYC (D) expression 72 hr after treatment of MT2 cells with parthenolide and ATO, alone or in combination. To note, relative expression in each treatment was compared with its relevant control; ATO (2 µM) with no treatment, parthenolide (1 µg/ml) with DMSO (0.2%), parthenolide (1 µg/ml) + ATO (2 µM) with DMSO (0.1%) + ATO (2 µM). * *P*<0.05,* * *P*<0.001, and * * * * P*<0.0001 compared with relevant controls

ATO: Arsenic trioxide; DMSO: Dimethyl sulfoxide

To unravel molecular mechanisms underlying the combinatorial effects of parthenolide and ATO, expression patterns of CD44*, NF-κB* (*REL-A*), BMI-1*, *and* C-MYC*, all of which contributed to proliferation and survival of ATLL cells, were studied by real-time PCR. As shown in Figure 3A, single-use of parthenolide and ATO significantly (*P*<0.0001) decreased CD44 expression in comparison with their relevant controls. Moreover, combination of parthenolide and ATO induced negative regulatory effects on CD44 expression when compared with DMSO and ATO treatment. Studying *NF-κB* (*REL-A*) expression revealed more interesting results; in comparison with relevant controls, parthenolide alone, but not ATO, significantly (*P*<0.05) down-regulated *NF-κB* (*REL-A*) expression and this effect was more considerable (*P*<0.001) when combination of parthenolide and ATO was used (Figure 3B). Similar changes were observed when BMI-1 expression was compared between each treatment and its relevant control. As presented in Figure 3C, single-use of parthenolide, quiet unlike ATO, significantly (*P*<0.001) down-regulated BMI-1 expression, and intriguingly, combination of parthenolide and ATO decreased BMI-1 expression to lower levels (*P*<0.0001). Nevertheless, analysis of *C-MYC* expression indicated a significant (*P*<0.001) down-regulation upon ATO treatment, and such alteration was not observed after administration of parthenolide alone or in combination (Figure 3D).

**Table 1:** List of primers, their sequence, and product length used for the real-time PCR analysis in current study

**Gene name**	**Sequence(5'→3')**	**Accession number**	**Product length bp**
***β***_2_ ***M***	**Forward**: AATTGAAAAAGTGGAGCATTCAGA **Reverse** : GGCTGTGACAAAGTCACATGGTT	NM_004048.3	127
***β***_2_ ***M***	**Forward** : TTGTCTTTCAGCAAGGACTGG **Reverse**: CCACTTAACTATCTTGGGCTGTG Probe: TCACATGGTTCACACGGCAGGCAT	NM_004048.3	127
***REL –A***	**Forward** : ACCCCTTCCAAGTTCCTATAGAAGAG **Reverse** : CGATTGTCAAAGATGGGATGAGAAAG Probe: ACTACGACCTGAATGCTGTGCGGCTCT	XM_011545206.2	145
***BMI-1***	**Forward** : CTGCAGCTCGCTTCAAGATG **Reverse** : CACACACATCAGGTGGGGAT	NM_005180.8	192
***C-MYC***	**Forward** : ACTCTGAGGAGGAACAAGAA **Reverse** :TGGAGACGTGGCACCTCTT	NM_002467.5	159
***CD44***	**Forward** : CGGACACCATGGACAAGTTT **Reverse** :GAAAGCCTTGCAGAGGTCAG	XM_006718390.4	176

**Table 2:** CI values for parthenolide and ATO combination calculated for different concentrations

Interpretation	CI	ATO (µM)	Parthenolide (µg/ml)
Synergism	0.79974	1	0.5
Synergism	0.80158	1.5	0.75
Synergism	0.71964	2	1

ATO: Arsenic trioxide; DMSO: Dimethyl sulfoxide

## Discussion

ATLL is an aggressive neoplasm of T-cells with serious complications in management and therapy, since resistance to conventional chemotherapeutics, both in the refractory stage and at the time of onset, is common among patients. Currently, designation of novel and more efficient combinatorial treatments is one of the main scopes in cancer research (18). The present study was carried out to investigate whether combination of parthenolide and ATO, a drug routinely prescribed for ATLL, could improve the efficacy of chemotherapy *in vitro*. 

Parthenolide is a natural terpenoid derivative with valuable anticancer activities. In the current study, assessment of MT2 cell viability revealed enhanced toxicity of ATO when used in combination with non-toxic parthenolide. Although previous studies have shown improved cytotoxicity of anticancer drugs doxorubicin, 5-fluorouracil, paclitaxel, cisplatin, and taxol by parthenolide in melanoma and colorectal and lung cancers (16, 19-21), this is the first report on efficient combination of parthenolide and ATO in hematologic cancer cells. Observed synergy in our study might be partially due to inhibitory effects of parthenolide on p-gp, an efflux pump that mediates drug resistance in ATLL (18, 22). 

Flowcytometry analysis revealed accumulation of MT2 cells in the sub G_1_ phase of the cell cycle upon ATO treatment, alone and especially in combination with parthenolide. In line with our findings, it has been demonstrated that parthenolide, in combination with 5-fluorouracil or tumor necrosis factor (TNF)-related apoptosis-inducing ligand (TRAIL), induced sub G_1_ arrest in colorectal cells (16, 23), similar to ATO that increased the proportion of myeloma cells in sub G_1_ phase (24). Since down-regulation of cell cycle promoters, for example, *Cyclin D1*, has been attributed to inhibitory effects of parthenolide (25), induced alterations in the MT2 cell cycle could be explained by the decreased level of such proteins.

In the investigation of mechanisms involved in the synergy between parthenolide and ATO, expression of CD44, *NF-κB* (*REL-A*), BMI-1*,* and *C-MYC* was analyzed by real-time PCR. CD44 is a transmembrane glycoprotein involved in metastasis and drug resistance of various cancer cell types(26). Overexpression of CD44 isoforms has been reported in hematopoietic malignancies such as non-Hodgkin’s lymphoma, myeloma, and chronic and acute myeloid leukemia. CD44 expression is also associated with undesirable clinical prognosis in lymphoma and myeloma(27-29). In ATLL patients, enhanced expression of CD44 is correlated with disease severity, and HTLV-1 oncoprotein Tax is involved in this de-regulation(30). We observed significant down-regulation of CD44 upon single and combinatorial administration of parthenolide and ATO in ATLL cells. Previous studies have demonstrated that overexpression of CD44 in acute lymphoblastic leukemia cells enhanced drug efflux and promoted chemotherapy resistance (26). Therefore, the synergy between parthenolide and ATO might be partially due to the negative effects of both chemicals on CD44 expression.

Members of the NF-κB family are involved in critical biological processes such as cell survival, cell cycle progression, and T-cell development (31, 32). In pathogenesis of ATLL, Tax activates NF-κB signaling, and thus, therapies that target the NF-κB pathway could induce apoptosis in ATLL cells (32). The negative effect of parthenolide on *NF-κB* expression has been previously reported in multiple myeloma and colon, gastric, and lung carcinoma cells (21, 33, 34). In addition, parthenolide in combination with balsalazide (prodrug of 5-aminosalicylate that reduces the risk of colon cancer in patients with ulcerative colitis) significantly suppressed nuclear translocation of NF-*κ*B in colon cancer cells and further *in vivo* studies in the murine model showed that this combination inhibited carcinogenesis (35). Moreover, it has been shown that parthenolide in combination with sulindac synergistically inhibited cell growth in pancreatic carcinoma cells, and this combination reduced transcriptional activities and DNA binding of NF-*κ*B (36). Similarly, our findings indicated a significant decrease in *NF-κB* (*REL-A*) expression by parthenolide, in single-use and also in combination with ATO. Since NF-κB promotes cell cycle by Cyclins D1, D2, D3, and E (37), decreased expression of *NF-κB* (*REL-A*) upon our combinatorial treatment could further explain sub G_1_ accumulation of MT-2 cells.

BMI-1 is an oncoprotein involved in cell cycle regulation and cell immortalization, and its up-regulation has been reported in acute myeloid leukemia (AML) and lung, ovarian, nasopharyngeal, and breast carcinomas (38, 39). Several studies have revealed the involvement of BMI1 in tumor cell invasion in gastric, hepatocellular, pancreatic, and endometrial carcinomas, as well as the correlation between BMI-1 up-regulation and chemotherapy resistance (40). Furthermore, overexpression of BMI1 was associated with drug resistance in hematological malignancies including myelodysplastic syndrome, chronic myeloid leukemia, AML, and lymphoma (41, 42). 

C-MYC is another oncoprotein that accelerates cell proliferation, and its de-regulated expression has been reported in various cancers such as osteosarcoma, glioblastoma, and melanoma, and breast, colon, cervical, and lung carcinomas (43). In addition, activation of C-MYC by oncoprotein Tax is associated with poor prognosis in acute and lymphomatous types of ATLL (44). 

For the first time, current findings indicated significant down-regulation of BMI-1 by parthenolide, and this effect was more considerable after our combinatorial treatment. Decreased expression of *C-MYC*, however, was only observed upon single-use of ATO, which is in line with reports that showed *C-MYC* down-regulation after ATO treatment of mantle cell lymphoma and lung cancer cells (45, 46).

## Conclusion

The present study provided evidence, for the first time, on combinatorial effects of parthenolide and ATO in ATLL cells. Since chemotherapy resistance is a major challenge in the treatment of ATLL, parthenolide could serve as a potent agent to improve the efficacy of current therapeutics. Despite great pharmacological properties of parthenolide, poor water-solubility of this compound caused limitations in clinical trials. Certainly, more research is necessary to clarify mechanisms of parthenolide action in different combinatorial strategies, before translating it to the clinical setting.
